# A Patient of Using Presepsin to Diagnose Streptococcal Toxic Shock Syndrome during Anticancer Drug Treatment

**DOI:** 10.1155/2019/3240501

**Published:** 2019-04-16

**Authors:** Gaku Takahashi

**Affiliations:** Department of Critical Care, Disaster and General Medicine, School of Medicine, Iwate Medical University, Japan

## Abstract

**Background:**

Streptococcal toxic shock syndrome (STSS) is a rapidly progressive infection, with potentially rapid patient deterioration in a very short period. We experienced a rare case of STSS during anticancer chemotherapy, and we continuously measured presepsin (P-SEP) and evaluated its usefulness.

**Case Presentation:**

A 60-year-old woman with pulmonary metastasis from cervical cancer began anticancer chemotherapy. A fever of >40°C and right lower leg swelling developed on day 3. Symptoms worsened despite cefmetazole treatment (1.0 g/day). Blood culture was performed without suspecting STSS. On day 5, symptoms worsened and acute disseminated intravascular coagulation (DIC) and sequential organ failure assessment (SOFA) scores increased. C-reactive protein (CRP) increased from 28.8 mg/dl to 35.5 mg/dl and P-SEP also increased from 1,635 to 2,350 pg/mL. STSS was suspected due to the rapid progression of brown discoloration of the entire right lower leg. Ceftriaxone 2 g/day and clindamycin 1,200 mg/day were begun. On the evening of day 5, blood culture revealed rapidly progressive group A streptococci. After that, symptoms improved rapidly with treatment, and SOFA and DIC scores also decreased. While CRP remained at about 0.5 mg/dl, P-SEP remained slightly elevated at about 400 pg/mL. A residual infection focus was suspected. Contrast-enhanced computed tomography (CT) revealed a capsule-enclosed abscess in the right lower leg soleus muscle on day 32. Debridement was performed and antibiotics were continued until P-SEP was 88 pg/mL. CT confirmed the disappearance of the abscess.

**Conclusion:**

Prompt diagnosis by blood culture and a sufficiently early, appropriate change in antibiotic therapy led to successful recovery from STSS during anticancer chemotherapy without lower limb amputation. P-SEP was useful in assessment of the residual infection focus and suspending treatments.

## 1. Background

Streptococcal toxic shock syndrome (STSS) is a sudden septic shock state caused by group A hemolytic streptococcus. The condition results in rapid multiple organ failure or death and is an extremely serious disease with a mortality rate of more than 30% [1, 2]. While in some cases, STSS is reported as a complication of other underlying diseases in middle or advanced age, or in immunocompromised patients, no underlying disease or significant medical history can be identified in 40% of cases. We encountered a female patient who suffered STSS during anticancer chemotherapy.

## 2. Case Presentation

**Figure 1:**
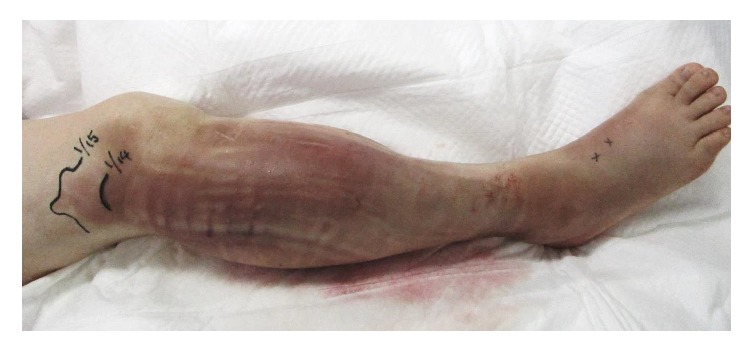
On day 6, the skin discoloration expanded to above the right knee.

**Figure 2:**
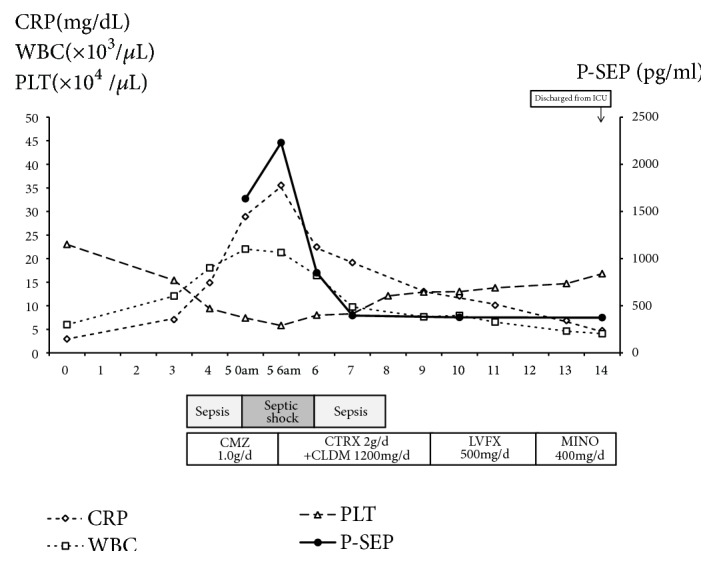
Time course of changes in P-SEP, WBC, CPR, and platelet count.

**Figure 3:**
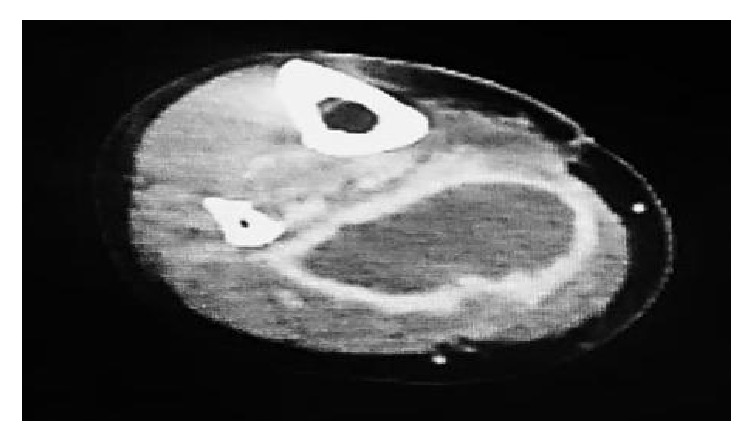
Contrast-enhanced CT on day 32.

**Figure 4:**
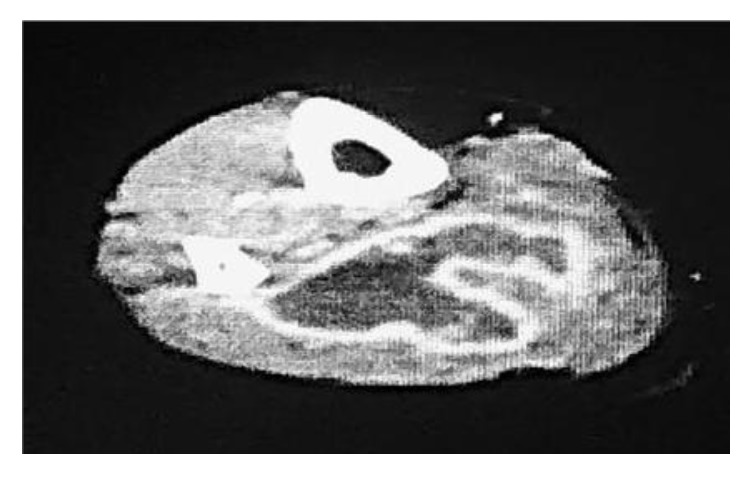
Contrast-enhanced CT on day 50; CT revealed a residual abscess.

**Figure 5:**
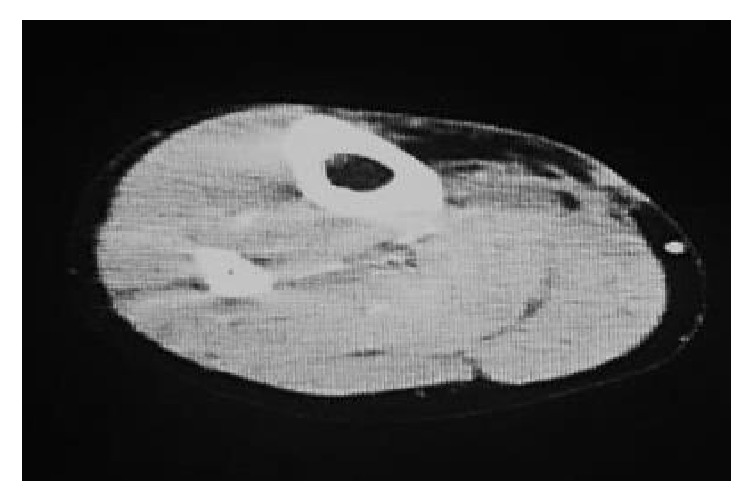
CT on day 100. Swelling was alleviated and disappearance of abscess was confirmed.

**Figure 6:**
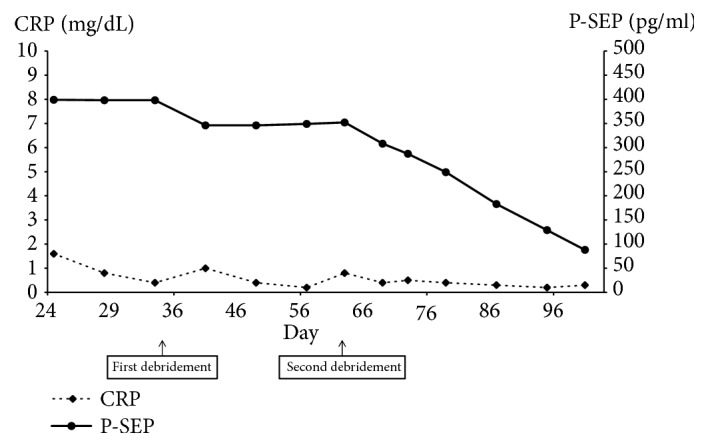
Time course of changes in P-SEP and CPR after the transfer to the general ward. Debridement was performed on day 34. Later, C-reactive protein (CRP) was 0.4–1.1 mg/dL, but P-SEP fluctuated between 350 and 380 pg/mL. On day 50, CT revealed a residual abscess. Therefore, we performed the second debridement on day 60. After that, P-SEP gradually decreased.

A 60-year-old woman was admitted to the gynecology ward at our hospital to undergo anticancer chemotherapy for pulmonary metastatic uterine cervical cancer. On day 1, she received cisplatin plus irinotecan infusions. On day 2, fever >40°C, diarrhoea, haematuria, and right lower leg swelling developed. Blood culture was performed, and cefmetazole (CMZ) 1.0 g/day was begun. On day 4, the right lower leg swelling worsened. A blood test revealed increased serum inflammatory markers, and acute disseminated intravascular coagulation [3] (DIC) and sequential organ assessment [4] (SOFA) scores increased to 6 and 7, respectively. The patient was transferred to our department on day 5 at 00:30 hours. At the initial examination at our department (Table 1), the serum CRP and P-SEP level were 28.8mg/dl nad 1,635 mg/mL, respectively. The Glasgow Coma Scale was 14, blood pressure 88/52 mmHg, and heart rate 90 beats/minute. Although severe swelling was observed in the posterior aspect of the right lower leg, there was no warmth or redness anywhere on the right lower leg. A 9-cm^2^ patch of brown skin discoloration was noted on the anterior surface of the tibia. Because the right popliteal artery was compressed significantly by the severe swelling in the lower leg soleus muscle, and the image quality was poor, no apparent abscess formation could be confirmed by contrast-enhanced computed tomography (CT) at this time. The popliteal vein was completely occluded, and deep venous thrombosis developed. To prevent potential progression to compartment syndrome, a relaxing incision was made on the medial right lower leg, and no distinct signs of infection were observed in the subcutaneous tissues or muscles. The patient was transferred to the intensive care unit (ICU), and nafamostat mesylate 150 mg/day and recombinant thrombomodulin 19,000 U/day were begun, along with low-molecular-weight heparin 15,000 E/day for venous thrombosis. At this point, STSS was not suspected, and CMZ was continued. A blood test on the morning of day 5 revealed a further exacerbation of the inflammatory markers, a further increase in the acute DIC score, and no improvement in the SOFA score. The brown discoloration progressed rapidly to the entire right lower leg. At this point, STSS was suspected for the first time, and antibiotic therapy was switched to ceftriaxone 2 g/day plus clindamycin (CLDM) 1,200 mg/day, and *γ*-globulin 15 g/day was initiated. On the evening of day 5, blood culture (in the day 3 specimen) was positive for rapidly progressive group A streptococci. After day 6, while the skin discoloration expanded to above the right knee (Figure 1), blood tests showed a trend towards improved. On day 14, the patient was transferred from the ICU to our general ward (Figure 2). The mild swelling and feverishness of the right lower leg continued. Contrast-enhanced CT on day 32 revealed an encapsulated abscess in the right lower leg soleus muscle (Figure 3). Debridement was performed with the patient under general anesthesia on day 34, close to the site of the relaxation incision. No organisms were isolated from the tissue culture of a specimen collected during debridement. After that, CRP remained at 0.4–0.6 mg/dL except during perioperative period, but P-SEP fluctuated between 350 and 380 pg/mL. We considered that local infections remained. And we continued to take oral minocycline (MINO) 200 mg/day. On day 50, CT revealed a residual abscess (Figure 4). Therefore, we performed the second debridement on day 60. After that, P-SEP gradually decreased and CT confirmed the disappearance of the abscess and swelling on day 100 (Figure 5). Oral MINO was discontinued after confirming that the P-SEP level had improved to 88 pg/mL (Figure 6). Subsequently, the patient was returned to the gynecological ward without recurrent infection.

## 3. Discussion

It is speculated that 5-12% of STSS infections are related to the medical environment [5, 6], especially related to surgery and childbirth [7]. In our case, the infection route could not be definitively identified; however, the patient presented with a 3-day history of sore throat before hospitalisation, and blood test at admission showed a slightly increased serum CRP of 2.8 mg/dL. Based on these findings, the infection was thought to be acquired via the pharyngeal mucosa, and the strain accumulated in the right lower leg muscle due to the patient's immunocompromised state.

Among the diagnostic criteria reported by the Centers for Disease Control and Prevention in 1993 [8], a positive blood culture, hypotension with systolic blood pressure <90 mmHg, and multiorgan failure were the factors for final diagnosis of STSS in our case.

In Japan, Nogami et al. [9] reported one case of STSS in 2014 in a patient with cervical cancer who suffered peritonitis after receiving cisplatin. In the same year, Fukumori et al. reported on a patient with hepatocellular carcinoma with soft tissue inflammation in the left thigh while receiving cisplatin and 5-fluorouracil chemotherapy as a Japanese paper. Nogami et al. [9] indicated the possibility of delayed STSS diagnosis in such patients due to influenza-like symptoms often observed as an adverse reaction of cisplatin infusion. Fukumori et al. also underscored the usefulness of early diagnosis using kits, because initial STSS symptoms often involve the upper respiratory tract, such as sore throat or fever, and some patients die before blood culture results become available. In our case, the blood culture specimen was collected on day 3, as soon as the high fever was noted, so as to obtain a definitive diagnosis at an early stage. In fact, if we had delayed switching the antibiotic therapy by a few days, the patient may have required lower extremity amputation or, worse, may not have been saved at all.

In addition to antibiotics, intravenous immunoglobulin (IVIG) was used to treat our patient. In the current sepsis guidelines [10], IVIG is not particularly recommended. However, Jessica et al. [11] reported that STSS treatment with IVIG and CLDM showed significant improvements in 28-day survival rates. In addition, Hamano et al. [12] compared single-dose administration of IVIG 15 g/day versus IVIG 5 g/day for 3 days and reported that single-dose IVIG 15 g yielded significantly greater improvements in features of systemic inflammatory response syndrome and serum interleukin-6 and lactate levels. We considered that administration of 5 g/day for 3 days is probably less effective because of the long half-life of IVIG (18–32 days). At our hospital, a single 15-g dose has been actively adopted. However, we acknowledge that improvement in just one case is not sufficient to demonstrate the efficacy of IVIG. In regard to the mechanism underlying the efficacy, Horstmann et al. [13] reported that group A streptococci suppress opsonization, which is potentially overcome, at least to some degree, by IVIG.

P-SEP is reported to be a novel infection marker indicating a higher diagnostic capability than CRP and also reflecting severity [14]. To our knowledge, this is the first report of the serial measurement of P-SEP in a case of STSS. In this case, the level of P-SEP increased with the exacerbation of symptoms and fell below the diagnostic cutoff level earlier than CRP with improved symptoms. However, there are reports that presepsin is affected by renal function [15, 16]. It has been confirmed that no decline in diagnostic accuracy was observed in patients with sepsis complicated with acute kidney injury [17], but in such patients it is considered that reexamination of the diagnostic cutoff value is necessary. On the other hand, in this case, P-SEP was very effective in the evaluation of the residual infection focus after AKI improved. The 95% confidence level of P-SEP for healthy persons is reportedly 314 pg/mL [13]. If the P-SEP level is >314 and <500 pg/mL, we think that some local infection may still remain. In fact, the right lower leg abscess remained as long as the P-SEP level was maintained around 400mg/dl, and the level decreased to 88 pg/mL after the abscess disappeared. During this period, CRP remained around 0.5 mg/dl and did not fluctuate so much, so it may be that P-SEP is superior in the evaluation of the residual infection focuses.

Because this patient was hospitalised, we could closely observe the course of disease progression, including changes in the skin lesion, over time. Furthermore, prompt diagnosis by blood culture, appropriate change of antibiotic therapy at a sufficiently early stage, and recurrence evaluation by P-SEP were effective in this case.

## 4. Conclusions

We experienced a rare case of STSS during anticancer chemotherapy. Prompt diagnosis by blood culture and appropriate change in antibiotic therapy at a sufficiently early stage led to successful recovery from STSS during anticancer chemotherapy without lower limb amputation. P-SEP was useful to evaluate the residual infection focuses.

## Figures and Tables

**Table 1:** Laboratory data at 0:30am on day 5.

Blood cell count		Blood biochemistry	

WBC	19.49×10^3^/*μ*L	AST	66 IU/l

Hb	12.4 g/dL	ALT	52 IU/l

Ht	34.7%	T-Bil	1.7 mg/dl

PLT	7.4×10^4^/*μ*L	*γ*GT	89 IU/l

Blood gas analysis		BUN	30.5 mg/dl

pH	7.440	Cr	1.56 mg/dl

PaO_2_	109.0 mmHg	CK	1514 IU/l

PaCO_2_	38.2 mmHg	Na	133 mEq/l

HCO_3_^−^	24.4 mmol/L	K	3.8 mEq/l

Lactate	2.4 mmol/L	Cl	100 mEq/l

Coagulation test		CRP	28.87 mg/dl

PT-INR	1.63	P-sep	1645 ng/dl

ARTT	41.5 s		

D-D	222.3 ng/mL	DICscore	6

ATIII	71%	SOFAscore	7

## Data Availability

The data generated and analyzed in this study are included in this published article and its additional files. The original datasets used for this study are not publicly available due to the existing regulation and only can be shared upon the approval of the directors of the corresponding hospitals.
